# Activating the genome during development and exit from mitosis

**DOI:** 10.1186/1756-8935-6-S1-O35

**Published:** 2013-03-18

**Authors:** Ken Zaret, Katherine Palozola, Jan Manuel Caravaca

**Affiliations:** 1 Department of Cell and Developmental Biology, Epigenetics Program, and Institute for Regenerative Medicine, Perelman School of Medicine, University of Pennsylvania 1056 BRB, 421 Curie Blvd, Philadelphia, PA 19104, USA

## 

During mitosis, the genome is silent. When cells exit mitosis and re-enter the cell cycle, how does the transcriptional program of a differentiated cell become reactivated faithfully and in a timely manner? We previously found that in undifferentiated progenitor cells in the early embryo, the competence to activate transcriptionally silent genes is established by pioneer transcription factors, such as FoxA proteins. Pioneer factors have the ability to bind their target DNA sequence in compacted chromatin and allow other factors to bind nearby. We discovered that the same pioneer transcription factors that function in early liver development remain bound to the genome during liver cell mitosis, when the genome compacts, most factors and RNA polymerase are excluded, and transcription is silent. Specifically, the pioneer transcription factor FoxA1 exhibits virtually complete mitotic chromosome binding, whereas other liver factors bind with a range of efficiencies and binding modalities. The evident hierarchy of specific binding, nonspecific binding, partial chromatin binding, failure to bind mitotic chromosomes, and mitotic instability reflects the factors’ developmental roles in gene activation, and suggest that re-activation of the genome following mitosis involves a replay of the network by which the cell type was established in development.

